# Prevalence of occupational stress in servants of a federal university

**DOI:** 10.47626/1679-4435-2023-829

**Published:** 2023-04-18

**Authors:** Deisyane Fumian Bouzada

**Affiliations:** 1 Programa de Pós-Graduação em Saúde e Nutrição, Universidade Federal de Ouro Preto, Ouro Preto, state of Minas Gerais, Brazil

**Keywords:** occupational health, occupational stress, universities, saúde do trabalhador, estresse ocupacional, universidades

## Abstract

**Introduction:**

Stress in the work context arises from situations in which the demands exceed the workers capacity to adequately respond to them or originate when the conditions offered and resources made available are insufficient to meet them.

**Objectives:**

To analyze the psychological demand, work control and social support among employees of a public university in the state of Minas Gerais.

**Methods:**

Quantitative, descriptive, and analytical epidemiological study. Data collection took place using an online questionnaire that included sociodemographic and occupational questions and the Demand-Control Model Scale, short version, including social support. Data were analyzed using the Stata version 14.0 program using descriptive and bivariate statistics.

**Results:**

The population consisted of 247 servants, including 49.2% teachers and 50.8% administrative technicians in education. In relation to gender, 59% were women and as to marital status, 51.8% were married. Regarding demand, 54.1% of workers had low demand, 59% had low control and 60.7% had low social support. The category of quadrants that included the largest number of servants was passive work with 31.2%. In the final model, the professional category variable maintained a significant association with occupational stress.

**Conclusions:**

The high prevalence of occupational stress (60.2%) and the low social support highlight the need for interventions so that these workers become agents of change in their work processes, being responsible for decisions made in their daily work

## Abstract (pt)

**Introdução:**

O estresse no contexto de trabalho decorre de situações em que as demandas excedem a capacidade de os trabalhadores respondê-las adequadamente ou origina-se quando as condições oferecidas ou os recursos disponibilizados são insuficientes para o seu atendimento.

**Objetivos:**

Analisar a demanda psicológica, o controle do trabalho e o apoio social em servidores de uma universidade pública mineira.

**Métodos:**

Estudo epidemiológico quantitativo, descritivo e analítico. A coleta de dados ocorreu por meio de questionário on-line, que incluiu questões sociodemográficas, ocupacionais e da escala do Modelo Demanda-Controle, acrescido do apoio social, versão resumida. Os dados foram analisados no programa Stata, versão 14.0, por meio de estatística descritiva e bivariada.

**Resultados:**

A população foi composta por 247 servidores, sendo 49,2% docentes e 50,8% técnicos administrativos em educação. Em relação ao sexo, 59,0% eram femininos, e, quanto ao estado civil, 51,8% eram casados. No que refere à demanda, 54,1% dos trabalhadores apresentaram baixa demanda, 59,0% apresentaram baixo controle e 60,7%, baixo apoio social. A categoria dos quadrantes que incluiu o maior número de servidores foi o trabalho passivo, com 31,2%. No modelo final, a variável “categoria profissional” manteve associação com significância para o estresse ocupacional.

**Conclusões:**

A elevada prevalência de estresse ocupacional (60,2%) e o baixo apoio social evidenciam a necessidade de intervenções no sentido de que esses trabalhadores se tornem agentes de mudanças nos seus processos de trabalho, sendo responsáveis pelas decisões tomadas no seu cotidiano de trabalho.

## INTRODUCTION

Stress is the state generated by the perception of stimuli that cause emotional excitement and, as a result of the disturbance of homeostasis, it triggers an adaptation process in which, among other changes, adrenaline and cortisol secretion is increased, leading to a number of systemic manifestations that, if experienced continuously, can lead to physiological and psychological disorders.^1^ In the work context, stress arises from situations in which the demands exceed the workers’ capacity to adequately respond to them or when the conditions offered and the resources available are insufficient to meet them, which is called occupational stress.^2^

Stress at work, or occupational stress, has been pointed out as an important risk factor in the development of negative outcomes to the worker’s health.^3^ One of the models internationally accepted by the academic community and widely used in scientific research related to the theme is the Demand-Control Model (DCM), plus social support. It studies the relationship between the psychological demands of the job and the worker’s decision latitude, mediated by the social support of co-workers.^4,5^ The Job Content Questionnaire (JCQ) is the instrument proposed to measure these dimensions.^4^ Based on three dimensions with 17 questions, the model makes it possible to understand the combination between work demands, decision-making autonomy, and social support in the work environment. Its main hypothesis is that adverse health reactions occur due to the psychological stress arising from the simultaneous exposure of workers to high psychological demands and little decision-making power over their work process (control) - highly demanding jobs.^4^ Social support from colleagues and bosses at work was added to the model,^6^ and its scarcity would be negative for health. Another hypothesis of the model would be a “positive effect” of stress, which could result in active behavior with motivation, new learning, and a positive coping pattern under simultaneous conditions of high psychological demands and decision span (active jobs). Conversely, the simultaneous shortage of psychological demands and decision span would lead to a state of demotivation, decreased learning, or even gradual loss of acquired skills (passive jobs).^6^

Due to their public sector employment, university workers are often undervalued, and viewed as inefficient, uninterested, and privileged because they have stable jobs.^7,8^ However, these workers may also be subjected to precarious working conditions, with levels of demand and control over their work that can lead to psychosocial stress at work, suffering, and disease.^8,9^

Considering that working and living conditions interfere in the worker’s health process, this study had the objective of analyzing the psychological demand, work control, and social support in employees of a public university in the state of Minas Gerais.

## METHODS

This was a quantitative, descriptive, and analytical (cross-sectional) research.

### DATA COLLECTION AND STUDY POPULATION

Data collection occurred using institutional e-mails of actively employed servants (professors and administrative technicians in education [ATEs]) of the Universidade Federal de Ouro Preto (UFOP) in the period from July to October 2020. Data were collected using an online semi-structured questionnaire developed on Google® Forms. The instrument included sociodemographic questions (gender, marital status, number of children, education, salary range, professional category, and time working at UFOP) and questions about the psychological demands, work process control, and social support of the Brazilian version of the abbreviated work stress scale. The abbreviated DCM scale contains 17 questions previously validated for the Brazilian Portuguese language, including five questions to assess demand, six questions to assess control, and six questions to assess social support.^10-12^

### STATISTICS

The occupational and sociodemographic characteristics were selected as independent variables, the DCM variables dichotomized into occupational stress with higher or lower exposure were selected as dependent or outcome variables. The higher exposure refers to all the servants with activities classified as highly demanding, passive work, and active work. The lowest exposure outcome comprises the servants who have activities classified as low demand.

To calculate psychosocial stress at work, we used the DCM quadrant formulation, which defines the following categories: low-demand work (low demand and high control), the reference group for psychosocial stress at work; passive work (low demand and low control), which can cause a reduction in the ability to solve problems in everyday work; active work (high demand and high control), which enables the worker to develop skills and abilities in their work; and high-demand work (high demand and low control), the highest exposure group for stress at work.^10,11,13,14^

The values for each dimension were obtained by summing the scores of the answers and then dividing them into two categories based on the median, according to the study by Alves et al.^11^ For demand, the cutoff point was 11 points; workers who scored between 5 and 11 points had their work classified as low demand, and those with values > 11, as high demand. For control, the cutoff point was 19 points; those individuals who obtained a total score between 6 and 19 points were classified as having low job control, and those with > 19 points, as having high job control. Similarly, the cutoff point for social support at work was 19 points, classifying individuals with scores ≤ 19 points as low social support and with > 19 points as high social support.^11^

Data were analyzed using Stata, version 14.0, including descriptive and bivariate analyses of socioeconomic, demographic, and occupational data and work characteristics, social support, and stress at work. Descriptive statistics of all study variables were presented using absolute and percentage frequencies. Bivariate analysis was also performed using the chi-square statistical test. The variables that presented statistical association in the univariate analysis (p-value ≤ 0.20) were selected for multivariate Poisson logistic regression (prevalence ratio [PR]) to verify their independent effect on the dependent variable (occupational stress). A significant association between the variables studied was considered when p value was ≤ 0.05, with a 95% confidence interval (95%CI).

### ETHICAL PROCEDURES

All ethical procedures of Resolution No. 466/2012 of the Brazilian National Health Council^15^ were respected in this study. The research received a favorable opinion from the Research Ethics Committee of UFOP. There was no conflict of interest on the part of the authors in conducting this research.

## RESULTS

Questionnaires were forwarded to 1,645 eligible servants during the data collection period (July to October 2020), with a response rate of approximately 15% (n = 247). Duplicate data were excluded. Among the participants, 49.2% were faculty and 50.8% were ATE. The most prevalent class of ATE were those with levels “D” and “E”, i.e., who hold professional technical level or high school and college education. Regarding gender, 59.0% were women, and as for marital status, 51.8% were married. As for the level of education, 85.0% had a graduate degree, 11.1% had completed higher education, and 3.9% had incomplete higher education. In relation to the number of children, 45.3% had no children, and 25.2% had only one. As for the economic classification, 51.7% reported receiving more than seven minimum wages, and 50.9% had at least 10 years in the institution.

Regarding demand, control, and social support at work, 54.1% of workers showed low demand, and 45.9%, high demand. Regarding control, 59.0% had low control, and 41.0% had high control. Most workers had low social support (60.7%) (Table 1).

**Table 1:** Characterization of the servants of the Universidade Federal de Ouro Preto according to psychological demands, control, and social support at work, dichotomized at the median distribution, Ouro Preto, 2021 (n = 231)^*^

Variables	Absolute frequency (n)	Relative frequency (%)
Demand		
Low	125	54.1
High	106	45.9
Control		
Low	138	59.0
High	96	41.0
Social support		
Low	142	60.7
High	92	39.3

* Differences in the n totals are due to loss of information for some variables.

Table 2 presents the distribution of servants in the quadrants of the DCM. The quadrant category that included the largest number of servants was passive work (low demand and low control), with 31.2%, followed by highdemand work (high demand and low control), with 28.1%. Low-demand work (low demand and high control) included 22.9% of the study population. The group with the lowest number of servants was the active workgroup, characterized by high demand and high control of work, with 17.7%. The overall prevalence of occupational stress (Table 2) in servants of the UFOP evaluated by the DCM was 60.2%. That is, 60.2% of the professionals experienced some stressful situation (of low control or high psychological demand), either simultaneously (both at the same time) or separately (one or the other of these situations). When assessing occupational stress by professional category, 62.3% of the teachers and 91.4% of the ATE reported its presence.

**Table 2:** Characterization of the servants at the Universidade Federal de Ouro Preto according to the quadrants of the Demand-Control Model and occupational stress, Ouro Preto, 2021 (n = 231)

Variables	Absolute Frequency (n)	Relative frequency (%)
DCM		
Passive work (↓D↓C)	72	31.2
Low demand (↓D↑C)	53	23.0
High demand (↑D↓C)	65	28.1
Active work (↑D↑C)	41	17.7
Occupational stress		
Yes	139	60.2
No	92	39.8
Total	231	100.0

C = control; D = demand; DCM = Demand-Control Model.

Passive work is more prevalent in women servants (64.0%), who do not have children (54.1%), with graduate level education (88.9%), whose salary range corresponds to more than seven minimum wages (55.5%), and with time at UFOP longer than 10 years (56.9%). Regarding the professional category, professors corresponded to 55.5%, and ATEs level “E” (higher level), to 56.2% among those of the same category. The highly demanding work differs from the passive work in relation to the professional category (prevalence of ATEs: 83.1%; and the salary range between two and four minimum wages: 43.1%) (Table 3).

**Table 3:** Characterization of servants of the Universidade Federal de Ouro Preto according to sociodemographic and occupational variables and quadrants of the Demand-Control Model, Ouro Preto, 2021 (n = 231)

	Low demand (↓D↑C)	Active work (↑D↑C)	Passive work (↓D↓C)	High demand (↑D↓C)
Sex				
Men	22 (9.6)	20 (8.7)	26 (11.2)	28 (12.1)
Women	31 (13.4)	21 (9.1)	46 (19.9)	37 (16.0)
Marital status				
Married	28 (12.1)	26 (11.2)	36 (15.6)	29 (12.5)
Civil union	11 (4.8)	5 (2.2)	13 (5.6)	5 (2.2)
Divorced	4 (1.7)	2 (0.9)	5 (2.2)	3 (1.3)
Widowed	1 (0.4)	0 (0.0)	0 (0.0)	0 (0.0)
Single	9 (3.9)	8 (3.5)	18 (7.8)	28 (12.1)
Number of children				
None	23 (9.9)	8 (3.5)	36 (16.9)	35 (15.2)
1 or 2	24 (10.4)	29 (12.6)	32 (13.9)	23 (9.9)
Above 3	6 (2.6)	4 (1.7)	1 (0.4)	7 (3.0)
Education				
Complete higher education	1 (0.4)	4 (1.7)	5 (2.2)	16 (6.9)
Incomplete higher education	0 (0.0)	1 (0.4)	3 (1.3)	5 (2.2)
Post-graduation	52 (22.6)	36 (15.6)	64 (27.8)	44 (19.0)
Salary range (minimum wages)				
1-2	0 (0.0)	1 (0.4)	0 (0.0)	1 (0.4)
2-3	1 (0.4)	3 (1.03)	3 (1.03)	10 (4.3)
3-4	1 (0.4)	3 (1.03)	9 (3.9)	18 (7.8)
4-5	1 (0.4)	0 (0.0)	2 (0.9)	7 (3.0)
5-6	3 (1.0)	4 (1.7)	6 (2.6)	5 (2.2)
6-7	6 (2.7)	6 (2.7)	12 (5.2)	8 (3.5)
> 7	41 (17.7)	24 (10.4)	40 (17.3)	16 (6.9)
Professional category				
ATE	10 (4.3)	21 (9.1)	32 (13.8)	54 (23.4)
Professors	43 (18.6)	20 (8.7)	40 (17.3)	11 (4.8)
Time at the UFOP (years)				
≤ 10	29 (12.6)	26 (11.3)	31 (1.4)	31 (1.4)
>10	24 (10.4)	15 (6.5)	41 (17.7)	34 (14.7)

ATE = administrative technicians in education; C = control; D = demand; UFOP = Universidade Federal de Ouro Preto.

The low demand job is more prevalent in women servants (58.5%); married (52.8%); with no children (43.4%); with a graduate level of education (98.1%); with a salary range higher than seven minimum wages (77.3%); and from the professors professional category (81.1%) (Table 3).

Active work is more prevalent in servants who were women (51.2%); married (63.4%); with one or two children (70.7%); with a graduate level of education (87.8%); with a salary range higher than seven minimum wages (58.5%); and in the ATE professional category (51.2%) (Table 3).

The prevalence of higher exposure to occupational stress related to socioeconomic variables was observed among single workers (PR = 1.12; 95%CI = 0.97-1.30) compared to workers of different marital statuses, and among those with an incomplete college education (PR = 1.04; 95%CI = 0.68-0.86), compared to the other levels of education. Regarding the occupational variables, employees within the ATE category have a higher risk of exposure to occupational stress (RR = 1.47; 95%CI = 1.26-1.71) compared to those who are professors.

The socioeconomic, demographic, and occupational variables and the outcome variable ‘occupational stress’ that maintained an association (p < 0.20) and were included in the final model were the following: marital status (p = 0.091); education (p = 0.009); salary range (p = 0.003); and professional category (p = 0.000). In the final model, only the variable “professional category” maintained an association with significance (p < 0.05) for the outcome variable (Table 4).

**Table 4:** Multivariate analysis of the occupational stress outcome - Poisson test, prevalence ratio, with 95% confidence interval, according to the explanatory variable, professional category among the civil servants of the Universidade Federal de Ouro Preto, Ouro Preto, state of Minas Gerais, 2021

Occupational stress	PR	95%CI	p-value
PROF_CAT	1.0		
21	1.53	1.24-1.87	0.00
22	1.55	1.33-1.81	0.00
23	1.44	1.14-1.81	0.02
24	1.46	1.23-1.73	0.00
25	1.41	1.18-1.67	0.00

95%CI = 95% confidence interval; PR = prevalence ratio

Table 5 shows the distribution of the study population according to the quadrants of the DCM and social support. The variable “social support” was significantly associated with the DCM quadrants (p = 0.023). The category that included the largest number of workers was passive work associated with low social support (n = 72; 35.25%). However, with regard to high social support, the category in the quadrants that included the largest number of workers was high-demand work (n = 34; 36.95%), which combines high demand and low control. The one that included the smallest number of workers was the low demand one (n = 14; 15.20%), which combines low demand and high control.

**Table 5:** Comparison between the variable “social support” and the quadrants of the Demand-Control Model and occupational stress in servants at the Universidade Federal de Ouro Preto, Ouro Preto, 2021 (n = 231)^*^

Category	Low social support (%)	High social support (%)	p-value
Low demand (↓D↑C)	39 (28.0)	14 (15.2)	0.023
Active work (↑D↑C)	20 (14.4)	21 (22.8)	
Passive work (↓D↓C)	49 (35.3)	23 (25.0)	
High demand (↑D↓C)	31 (22.3)	34 (37.0)	
Total	139 (100.0)	92 (100.0)	
Occupational stress			
Yes	100 (71.9)	39 (28.1)	
No	78 (84.8)	14 (15.2)	

* Chi-square test.

C = control; D = demand.

## DISCUSSION

The data showed that there was a predominance of women (59%) among the workers in the population studied. Besides working in paid activities, women dedicate part of their time to domestic activities and caring for children and other family members, which can generate an overload of activities for these workers.^16^ Regarding the professional category, there was a proportional distribution between ATEs and professors (50.8 and 49.2%, respectively), although the difference was small between them. As for marital status, approximately 66% are married or in a stable union; however, 45.3% declared not to have any children.

Regarding the level of education, 85% of the servants had a post-graduation degree, This can be explained by the fact that they work in a public university, have job stability, and can compete for the percentage of vacancies assigned to public servants in graduate courses in this institution, generating a stimulus for their insertion in the training process. A study conducted with administrative technicians of a state higher education institution revealed that improving the level of education of the worker is a positive aspect of the quality of life at work. However, when the worker’s level of education is higher than that required for his or her position, as well as when his or her payment is not compatible with his or her level of education, it is possible that this causes demotivation, drop in productivity, conflicts with the boss, among other aspects.^17^

The category of the DCM quadrants that included the largest number of people was passive work (31.2%), which combined low demand and low control, being considered the second most problematic exposure for health and consequently for occupational stress. The first is the high-demand work, which combines high demand and low control, which, in the servants of the sample, presented a frequency of 28.1%. These two types do not allow the worker to grow, leading to a gradual atrophy of learning skills.^11,18^ Similar results were found in other universities, such as the research conducted with ATE workers at the Universidade Federal de Juiz de Fora, which found the highest number of workers (39.7%) also in passive work. Alves et al.^19^ also showed, in a similar population (women in the Pro-Health Study), a high prevalence of passive work (28.3%), followed by high demand (24.8%) and low demand (20.9%). In another study conducted with administrative technical workers of a federal university in the South of Brazil, it was found that 28% of the ATE performed passive work and 27% low-demand work.^20^

Although there is a low association between the worker’s income and the presence of occupational stress, this study showed that the workers with better income are less prone to occupational stress, suggesting the importance of this variable in workers’ health, making it possible to face situations of high demand and low control, a category of greater exposure to occupational stress (high demand).

Several DCM studies have been conducted with workers, mainly health professionals, with evidence of an association between work-related stress and several outcomes, such as high blood pressure^20^, coronary heart disease,^21^ obesity,^22^ mental disorders,^21^ among others. A survey of the UK Workforce^23^ suggests that stress is the leading cause of work-related illness, accounting for about 40% of all new incidences of sickness absence. In another study, conducted in Norway, a significant association was found between work stress and long-term sick leave.^24^

The low control over the work process in association with low demand (passive work) can be a discouraging factor, contributing to an increase in work dissatisfaction^25^ and, consequently, to a drop in the individual’s production, besides the decrease in the capacity to produce solutions for the activities and problems faced in the daily work routine.^10^

Regarding the outcome evaluated, measured by the DCM, the results showed a very high frequency of occupational stress in the studied population. More than 60% of the studied group was exposed to situations of high psychological demand or low level of control over their work, simultaneously or separately. Similar results were found in other studies. The global prevalence of occupational stress among nursing workers in medium complexity services in the state of Bahia, evaluated by DCM, was 77%.^26^ Another study, conducted in the state of Mato Grosso do Sul, showed prevalences of occupational stress of 70%,^27^ similar to those found in this study, evidencing the severity of the outcome. Stress in the work environment stems from aspects of the organization, administration, and work system as well as the quality of human relations.^28^

Social support is an essential psychosocial aspect in all work sectors. It allows individuals, in their daily work, to increase their ability to solve the problems presented by users and by the dynamics of the service itself, besides developing solidarity when faced with adverse situations. Moreover, it allows the sharing of mutual commitments.^29^ The low social support among UFOP employees (60.7%) is rather worrisome, and it can impact the health of individuals. In an investigation with hospital workers (public servants) using the DCM, social support, and burnout to identify the duration of absenteeism and health treatment leaves (HTL), there was an increase of 2.04 in the expectation of HTL days due to low support from colleagues, which presumes that, regardless of being related to a passive work or not, low social support can contribute to illness.^30^

The Whitehall II study found that British civil servants with frequent occupational stress and low social support at work were more likely to experience major depressive disorder (MDD), which indicates the relevance of social support at work.^21^

## CONCLUSIONS

When analyzing the psychological demand and work control of the university’s servants, it was possible to identify that the category of passive work predominated. The high prevalence of occupational stress and the low social support show the need for interventions so that these workers become agents of change in their work processes, being responsible for the decisions made in their daily work. Stress is a worldwide concern, which affects populations of several cultures with the most different work organizations. In relation to occupational stress, that is, the stress that affects the worker in his/her production relations, there are few studies conducted in public educational institutions in Brazil. The workers have been adjusting to the reformulations in the models of management and organization in the world of work, which also happen in public institutions. The institution must therefore invest in research to understand the relations of the illness process since the public servant is the main agent of this process.

The positive points of this study were the sociodemographic heterogeneity, which contributed to the variability of the social determinants of the population. Another positive point related to the population of this study is the variety of occupations performed since it is appropriate for the theoretical model used in this research that aimed to investigate psychosocial stress at work. As a negative point, there was difficulty in data collection due to the onset of the COVID-19 pandemic, which made it impossible to collect data from servants who did not access social networks and institutional e-mails.
